# Domestic

**Published:** 1841-01-09

**Authors:** 


### DOMESTIC.

*Introductory Lectures.*—We acknowledge the receipt of introductory lectures by Professors Harvey Lindsley and Thomas Miller, of the Columbian College, Washington, D. C. We hope, at an early opportunity, to notice them at some length.

---

*Copying without credit.*—The Boston Medical and Surgical Journal has pounced upon an accidental omission on the part of the Examiner to credit it for Dr. Dix's cases of strabismus, and makes considerable stir thereupon. We regret the omission, and believe that our previous character is a sufficient guarantee that it was unintentional. A propos of "editorial courtesy," "fair play," and "giving credit for whatever is borrowed," will our Boston friend inform us, if the articles quoted from *Bul. de l'Academ.*, *Graefe's and Walter's Journal*, *Observatore Medico*, and *Bibliothek fur Laeger*, in the number of his Journal in which the Examiner is berated, were *translated* and copied directly from the sources to which they are ascribed.

---

*Connecticut Retreat for the Insane.*—From the report for this institution for the year 1840, we extract some details which will be read with interest in our own and other states which have not yet followed the example of New England in providing for their insane poor.

"It is now sixteen years since the Connecticut Retreat was opened for the reception of patients.

From the reports of the physicians, it appears that on the first of April, 1840, one thousand patients had been admitted into the Retreat. About five hundred of these were labouring under some form of insanity of recent date, and brought to the institution with the expectation of benefit from the means employed for their recovery. Of this number, four hundred and fifty have been restored to reason and returned to their families, and to those stations of usefulness which they had occupied before their attack.

Of the five hundred cases of chronic insanity, a large number has been cured; many have left the institution much improved, and others remained, not for the purposes of medication, but because they were made more comfortable there than they could possible have been at their own homes. Even of this latter class—the forlorn hope of the institution—there have been some happy recoveries occurring, two, five, or ten years after the attack, and when all expectation of such relief had been obliterated from the minds of their relatives and guardians. Those who have witnessed these results, and those who have experienced these benefits, are among the warmest and best friends of the institution."

Dr. A. Brigham has been elected superintendant of the Retreat—an appointment well spoken of.

---

*Philadelphia Medical Society.*—At the annual election held on Saturday, 2d January, the following officers were elected for the ensuing year:—

*President.*
Thomas Harris, M. D.
*Vice Presidents.*
George B. Wood, M. D.
Robert M. Huston, M. D.
*Treasurer.*
J. Brewer, M. D.
*Corresponding Secretaries.*
B. H. Coates, M. D.
W. Poyntell Johnston, M. D.
*Recording Secretary.*
Joshua M. Wallace, M. D.
*Orator.*
Isaac Parrish, M. D.
*Librarian.*
J. F. White, M. D.
*Curators.*
Joseph Peace, M. D.
Jno. B. Griscom, M. D.

---

*Medical Prize Questions for* 1841.—*New York Prize Questions.*—The sum of fifty dollars is offered by the Medical Society of the State of New York, to the successful candidates on each of the following questions:

1st. The medical literature of *Cholera Morbus*, previous to the appearance of the epidemic Cholera. [*Note. It is expected that the medical history of Cholera Morbus in this country will be particularly examined.*]

2d. An analysis of the discoveries concerning the *Physiology of the Nervous System*, from the publications of Sir Charles Bell to the present time, both inclusive. Dissertations to be directed to Peter Van Olinda, M. D. Secretary, Albany, N. Y.

*Boylston Prize Questions.*—1st. To what extent is disease the effect of changes in the chemical or vital properties of the blood?

2d. The structure and diseases of the teeth, with a numerical solution of the question—can caries of the teeth be retarded by mechanical processes?

Dissertations on these subjects must be transmitted, post paid, to J. C. Warren, M. D., Boston, on or before the first Wednesday of April, 1841.

The following questions are offered for 1842. 1st. To what extent is the human system protected from small pox, by inoculation with the cow pox? Is the protection increased by revaccination; and, if so, under what circumstances?

2d. On the diseases of the kidney, and the changes which occur in the appearance and composition of the urine, in health and in disease.

Dissertations on these questions must be transmitted as above, on or before the first Wednesday of April, 1842.

The author of the best dissertation on either of the above subjects, will be entitled to a premium of fifty dollars, or a gold medal of that value, at his option.

Each dissertation must be accompanied by a sealed packet, on which shall be written some device or sentence, and within shall be enclosed the author's name and residence. The same device or sentence is to be written on the dissertation to which the packet is attached.

All unsuccessful dissertations are deposited with the Secretary, from whom they may be obtained, if called for within one year after they have been received.

By an order adopted in 1826, the Secretary was directed to publish annually the following votes, viz.:

1st. That the Board do not consider themselves as approving the doctrines contained in any of the dissertations to which the premiums may be adjudged.

2d. That in case of the publication of a successful dissertation, the author be considered as bound to print the above vote in connection therewith.

*Fiske Medical Prize Questions for* 1841.—The sum of fifty dollars will be awarded by the Medical Society of Rhode Island, to the author of the best dissertation on either of the following subjects, or a gold medal of equal value, at the option of the successful competitors.

1st. Spinal diseases, structural and functional. 2. Dropsy, its causes, nature, and treatment. Dissertations are to be sent free of expense, to R. Brownwell, M. D., Providence; T. C. Dunn, M. D., Newport, or J. Williams, M. D., Warren.—*American Medical Almanac.*

